# Surgical Masks Affect the Peripheral Oxygen Saturation and Respiratory Rate of Anesthesiologists

**DOI:** 10.3389/fmed.2022.844710

**Published:** 2022-04-14

**Authors:** Shaozhong Yang, Chuanyu Fang, Xin Liu, Yu Liu, Shanshan Huang, Rui Wang, Feng Qi

**Affiliations:** Department of Anesthesiology, Qilu Hospital of Shandong University, Jinan, China

**Keywords:** anesthesiologists, heart rate, peripheral oxygen saturation, respiratory rate, surgical mask

## Abstract

**Background:**

Surgical masks (SMs) protect medical staff and reduce surgical site infections. Extended SM use may reduce oxygen concentrations in circulation, causing hypoxia, headache, and fatigue. However, no research has examined the effects of wearing SMs on oxygenation and physical discomfort of anesthesiologists.

**Methods:**

An electronic questionnaire was established and administered through WeChat, and a cross-sectional survey was conducted to determine SM use duration and related discomfort of operating room medical staff. Then, operating room anesthesiologists were enrolled in a single-arm study. Peripheral blood oxygen saturation (SpO_2_), heart rate, and respiratory rate were determined at different times before and after SM use. Shortness of breath, dizziness, and headache were subjectively assessed based on the visual analog scale (VAS) scores.

**Results:**

In total, 485 operating room medical staff completed the electronic questionnaire; 70.5% of them did not change SMs until after work, and 63.9% wore SMs continuously for more than 4 h. The proportion of anesthesiologists was the highest. After wearing masks for 4 h, the shortness of breath, fatigue, and dizziness/headache rates were 42.1, 34.6, and 30.9%, respectively. Compared with other medical staff, the proportion of subjective discomfort of anesthesiologists increased significantly with prolonged SM use from 1 to 4 h. Thirty-five anesthesiologists completed the study. There was no difference in anesthesiologist SpO_2_, heart rate, or respiratory rate within 2 h of wearing SMs. After more than 2 h, the variation appears to be statistically rather than clinically significant—SpO_2_ decreased (98.0 [1.0] vs. 97.0 [1.0]*, p* < 0.05), respiratory rate increased (16.0 [3.0] vs. 17.0 [2.0]*, p* < 0.01), and heart rate remained unchanged. As mask use duration increased, the VAS scores of shortness of breath, dizziness, and headache gradually increased.

**Conclusion:**

In healthy anesthesiologists, wearing SMs for more than 2 h can significantly decrease SpO_2_ and increase respiratory rates without affecting heart rates.

## Introduction

Since December 2019, coronavirus disease 2019 (COVID-19) has spread globally and attracted worldwide attention (1). On 11 March 2020, the World Health Organization (WHO) declared COVID-19 a pandemic (2). It is essential to protect healthcare workers in the context of the COVID-19 pandemic. Medical institutions require medical staff to wear personal protective equipment during diagnosis and treatment (3). However, frequent use of personal protective equipment, especially protective masks, can lead to various health-related problems, including headache, hypoxia, and skin damage (4, 5).

In daily work, anesthesiologists face long exposures to infected bodily fluids and blood, toxic surgical smoke, bacteria, and viruses in patients' respiratory tract and the possibility of needle stab injuries, resulting in anxiety and fear (6–8). As part of the main staff in the operating room, anesthesiologists must follow the recognized infection control measures for the operating room, including the use of surgical masks (SMs). Although there is still substantial controversy (9, 10), it is generally believed that wearing SMs can protect patients from bacteria in the mouth and nose of operating room staff and protect medical staff from contaminated blood, bodily fluids, and most of the operation smoke (11).

At present, there is a serious shortage of anesthesiologists in China. The number of sudden deaths of anesthesiologists in China has increased sharply in recent years, and most of them die due to heavy workloads (12). From 11 November 2008 to 27 January 2018, 104 deaths due to overwork were reported among Chinese doctors, and anesthesiologists accounted for 20.19% of these cases (13). Increasing work pressure often leads to excessive fatigue and physical discomfort in anesthesiologists and threatens the safety of patients (14, 15). Chinese anesthesiologists need to wear SMs to work for long periods of time every day, which may increase physical and mental fatigue. Research shows that wearing masks can significantly increase the temperature and humidity of the face, resulting in a decline in the mental and physical performance of medical staff (16, 17). Recent studies have confirmed that SMs can reduce blood oxygen saturation and increase the pulse rate in surgeons after surgery (18). Although wearing SMs during vigorous exercise had no significant adverse effects on the oxygenation and exercise performance of young healthy participants (19), there is no relevant study on the effects of SM use on anesthesiologists' oxygenation and physical discomfort.

It is unclear whether wearing SMs for a long time causes changes in anesthesiologists' oxygenation, heart rate, and respiratory rate and aggravates physical and mental fatigue and whether wearing SMs for a long time is related to the high rate of sudden death among Chinese anesthesiologists. The primary purpose of this study was to investigate the effects of wearing disposable lace-up SMs for long periods of time on peripheral blood oxygen saturation (SpO_2_), heart rate, respiratory rate, and subjective discomfort of anesthesiologists.

## Materials and Methods

This study was carried out in accordance with the Declaration of Helsinki (2013 Edition) and approved by Qilu Hospital of Shandong University Medical Ethics Committee (No. KYLL-202107-115). The requirement to obtain written informed consent was waived.

First, an electronic questionnaire was designed on the Wenjuan platform (https://www.wenjuan.com) and distributed *via* WeChat in 8 general hospitals in Shandong Province. The duration of mask use and the related discomfort of medical staff in the operating room were determined by a cross-sectional survey. The survey included demographic data (gender, occupation), the reasons for wearing SMs in the operating room, the duration of continuous mask use, the number of mask changes per day, and personal discomfort related to continuous mask use for 1, 2, 3, and ≥4 h. After the questionnaire was released, participants were asked to familiarize themselves with the questions and reply to the questionnaire after 1 week of work. Only one submission per person was allowed to prevent duplication.

Then, according to the survey results, anesthesiologists working in the operating room of Qilu Hospital of Shandong University were selected as subjects. They were required to wear disposable lace-up SMs (the Chinese standard number is YY0469-2011) correctly and not remove them or expose their mouth and nose during the study. All subjects were informed that they could withdraw from the study at any time without any impact. The primary exclusion criteria were acute or chronic respiratory diseases or previous heart diseases, body mass index (BMI) ≥30 kg/m^2^, the presence of inflammation on the facial skin, lax skin, allergic rhinitis, and nasal septum deviations.

The enrolled anesthesiologists fully understood the study procedures, underwent testing in a familiar constant temperature operating room, and breathed indoor air. The subject ensured that the SM was correctly and continuously worn throughout the process. After resting for at least 10 min, the SpO_2_ and pulse rate of the subject's right index finger were measured using a portable SpO_2_ patient monitoring system (Covidien LLC, Manstield, OH, USA) in a sitting position. To eliminate the difference in oxygen content in the operating room, an oxygen detector (Smart Sensor, Dongguan, China) was used to detect the surrounding oxygen content. Another researcher also directly observed 30 s of chest movement and measured the respiratory rate. As a distraction strategy, the investigators pretended to measure the radial pulse so that participants did not realize that their respiratory rate was being measured.

Participants underwent pulse oximetry evaluation. According to the questionnaire survey results, it was determined that data would be collected at 6 time points: 10 min before wearing an SM (T1), immediately after wearing a mask (T2), and after 1 h (T3), 2 h (T4), 3 h (T5), and 4 h (T6) of continuously wearing an SM. The visual analog scale (VAS) was used to record subjects' perception of the sensation of shortness of breath, dizziness, and headache at the same time points. All sensations were scored by means of a 10-point VAS from 0 (no discomfort) to 10 (worst discomfort imaginable). To minimize variability, data were collected twice at each point in time.

### Statistical Analysis

The study sample size was calculated with PASS 11 (NCSS, LLC Kaysville, UT, USA). Before the release of the electronic questionnaire, we conducted a small-scale preliminary survey. The incidence of wearing SMs continuously for morethan 4 h was 55%. We assumed that the incidence was 50%, with α of 5%, absolute deviation of 5%, and the sample size of 402. Previous studies have shown that masks can reduce the blood oxygen saturation of medical staff by 1–3.5% (20, 21). We determined whether wearing an SM was associated with an SpO_2_ decrease of 2% or more. For a 2% decrease in SpO_2_, a standard deviation of 3%, α of 5%, and power of 90%, a sample size of at least 24 participants was required. Considering the 10% shedding rate, at least 27 participants were required. According to the inclusion and exclusion criteria, we included 39 anesthesiologists. One participant whose resting oxygen saturation reading was lower than 96% and 3 participants who did not complete the test due to removing their masks were excluded, and a total of 35 subjects were included.

The result of electronic questionnaire was charted by Microsoft Excel software. The SpO_2_, heart rate, respiratory rate data, and subjective sensation scores of anesthesiologists wearing SMs at different durations were analyzed. SPSS 24.0 (IBM, Armonk, NY: IBM Corp.) Graphpad prism 8.2.1 (GraphPad Sofware Inc., California, US) were used for the statistical evaluation and preparation of graphs.

The Shapiro–Wilk test was used to determine whether the measurement data conform to the normal distribution. The data with normal distribution were expressed as mean (standard deviation, SD), and the non-normal distribution data were expressed as median (interquartile range, IQR). Univariate ANOVA was used for comparisons between groups. The non-normal distribution data were analyzed by the Kruskal–Wallis test. A *p* value of < 0.05 was considered statistically significant.

## Results

### Occupation and Gender of Operating Room Medical Staff Participating in the Electronic Questionnaire

**Figure 1:**
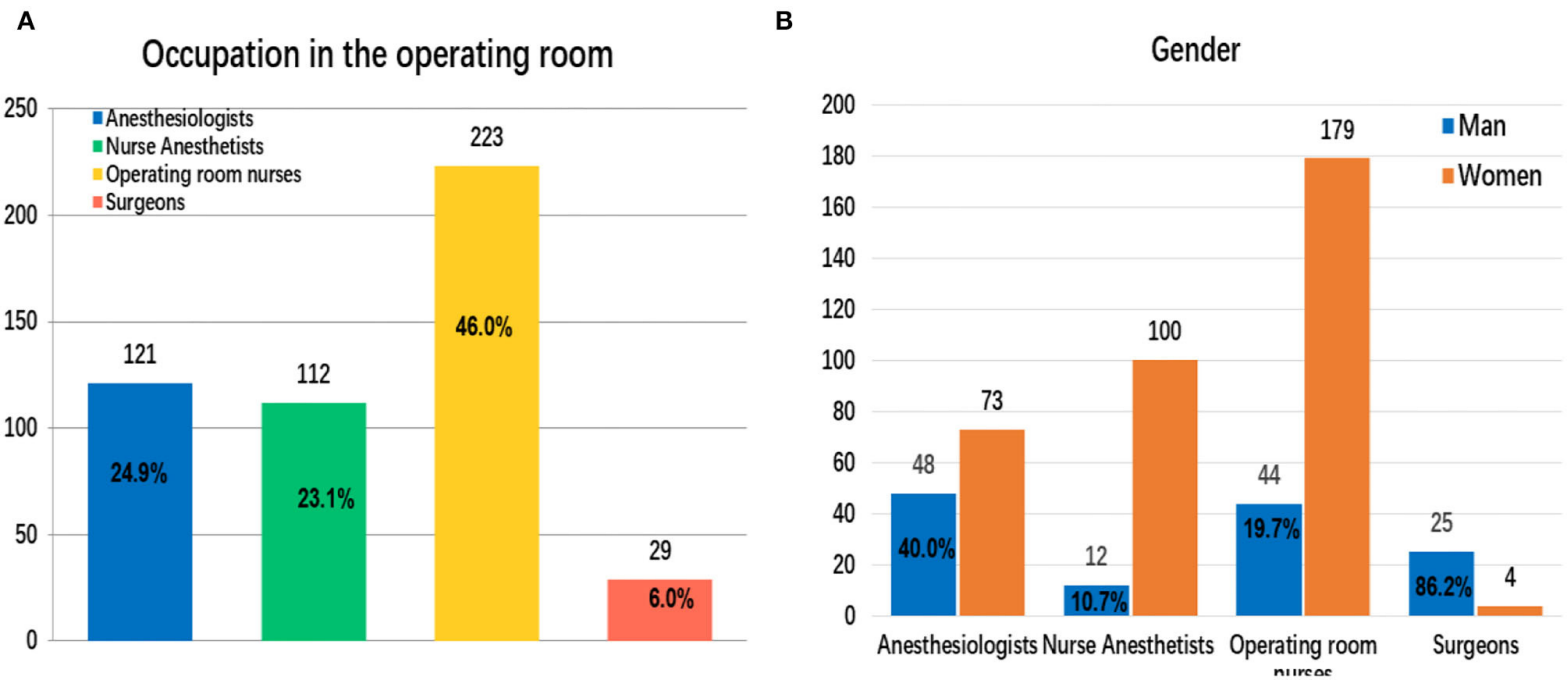
Occupation and gender of operating room medical staff who participated in the electronic questionnaire **(A,B)**.

A total of 485 operating room medical staff completed the electronic questionnaire survey, of which anesthesiologists accounted for 24.9% (*n* = 121/485) (Figure 1A). Due to professional reasons and gender factors, the gender difference of anesthesiologists was smaller, and men account for 40.0% (*n* = 48/121) (Figure 1B). As shown in Figure 1B, compared with anesthesiologists (48 men [40.0%]), there were greater gender differences among operating room nurses (42 men [19.7%]), nurse anesthetists (12 men [10.7%]), and surgeons (25 men [86.2%]), and surgeons had lower participation in the questionnaire.

### Duration of Continuously Wearing SMs and the Frequency of Mask Changes on Working Days as Reported *via* the Electronic Questionnaire

**Figure 2:**
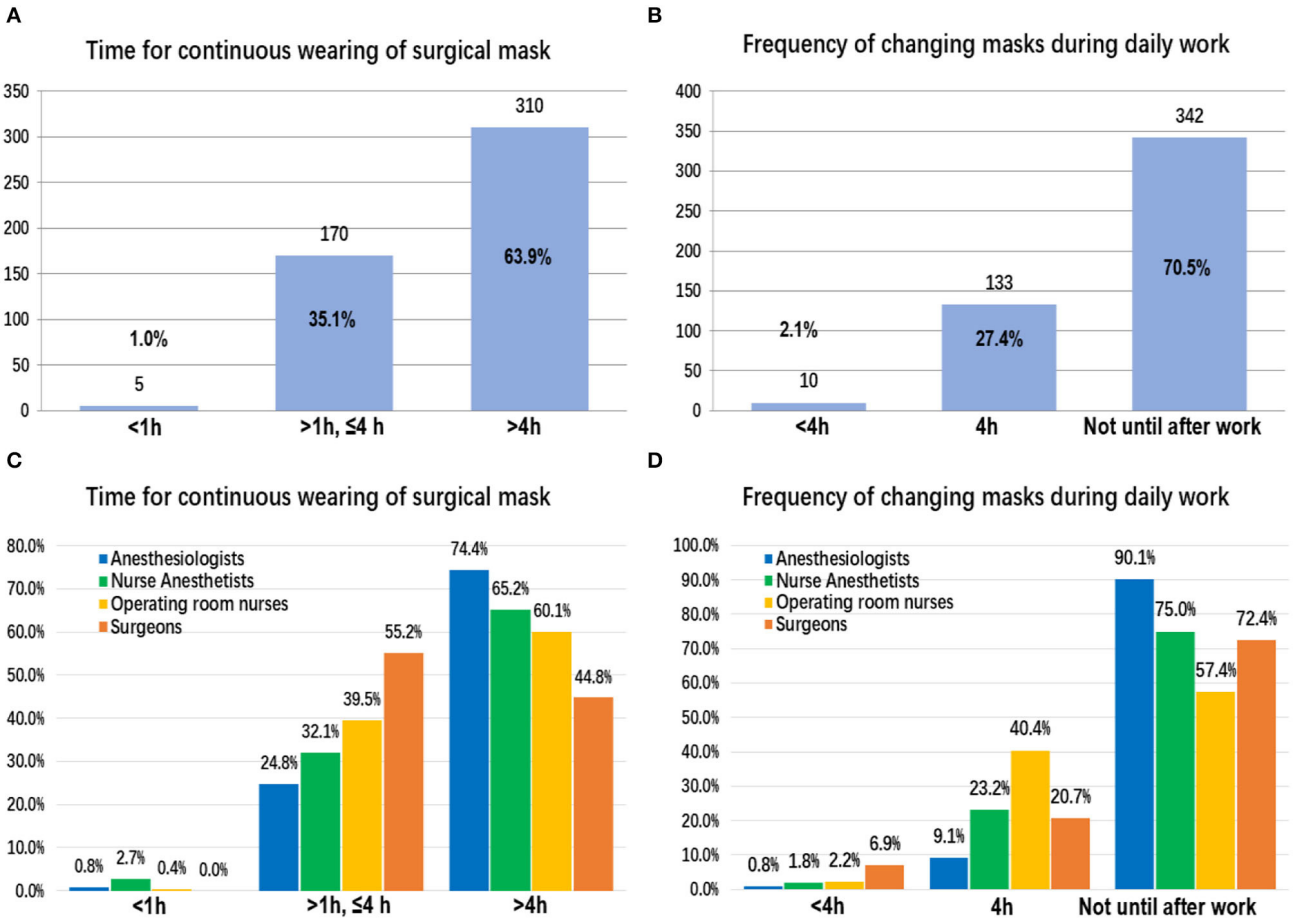
The duration of continuous surgical mask use and the frequency of mask changes on working days: among all participants **(A,B)** and between different occupations **(C,D)**.

A total of 63.9% (*n* = 310/485) of the participants wore SMs continuously for more than 4 h (without removing the masks or exposing their mouths or noses) (Figure 2A). Among the four occupations, the proportion of anesthesiologists wearing SMs continuously for more than 4 h was highest, which was 74.4% (*n* = 90/121) (Figure 2C). On a given day of work, 70.52% (*n* = 342/485) did not change their mask until after work (Figure 2B). Among them, the proportion of anesthesiologists was still the highest, accounted for 90.1% (*n* = 109/121) (Figure 2D).

### Subjective Discomfort of Wearing Masks for Different Duration as Reported in the Electronic Questionnaire

**Figure 3:**
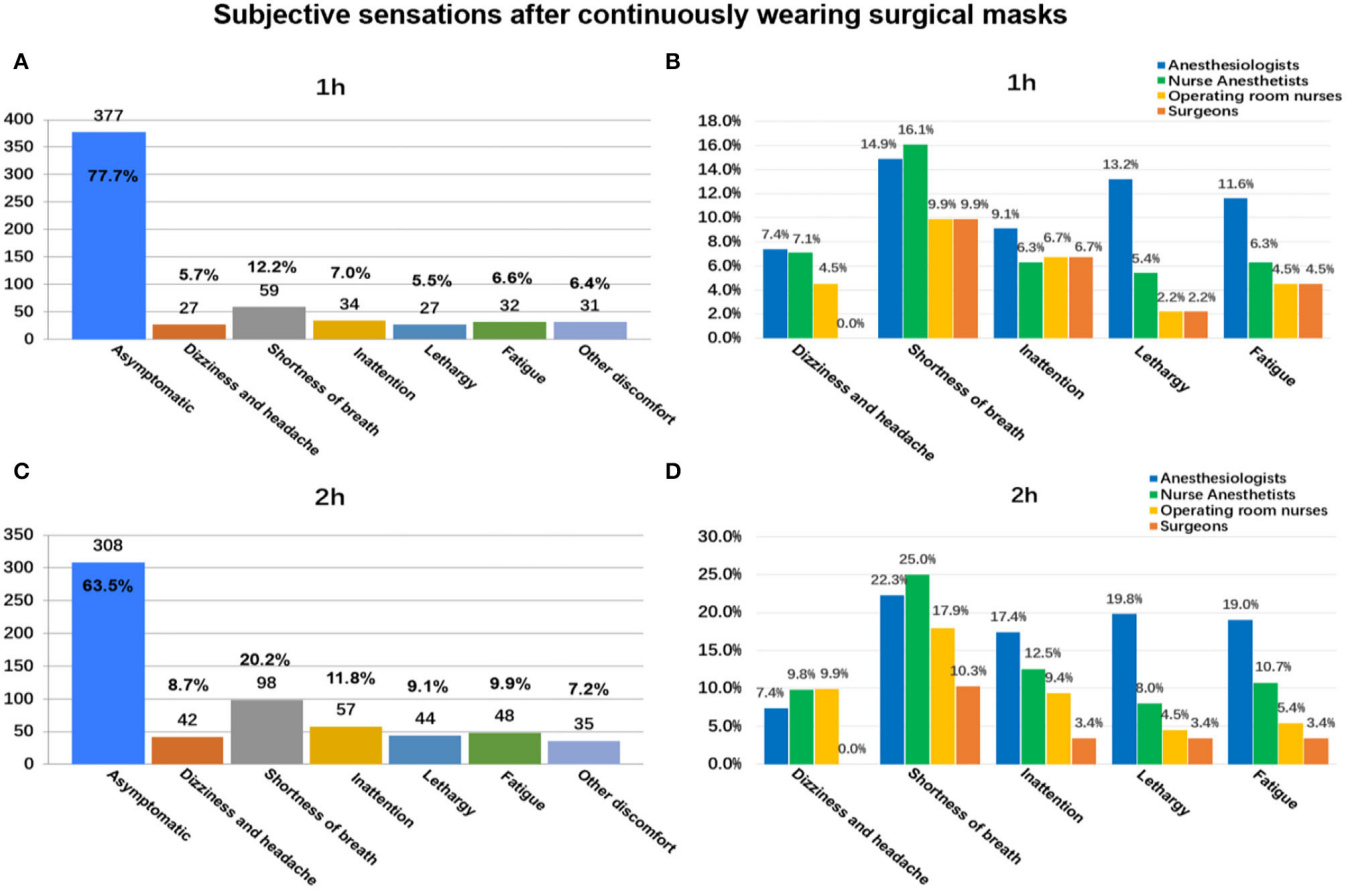
Subjective discomfort associated with continuously wearing surgical masks for 1 and 2 h: among all participants **(A,B)** and between different occupations **(C,D)**.

**Figure 4:**
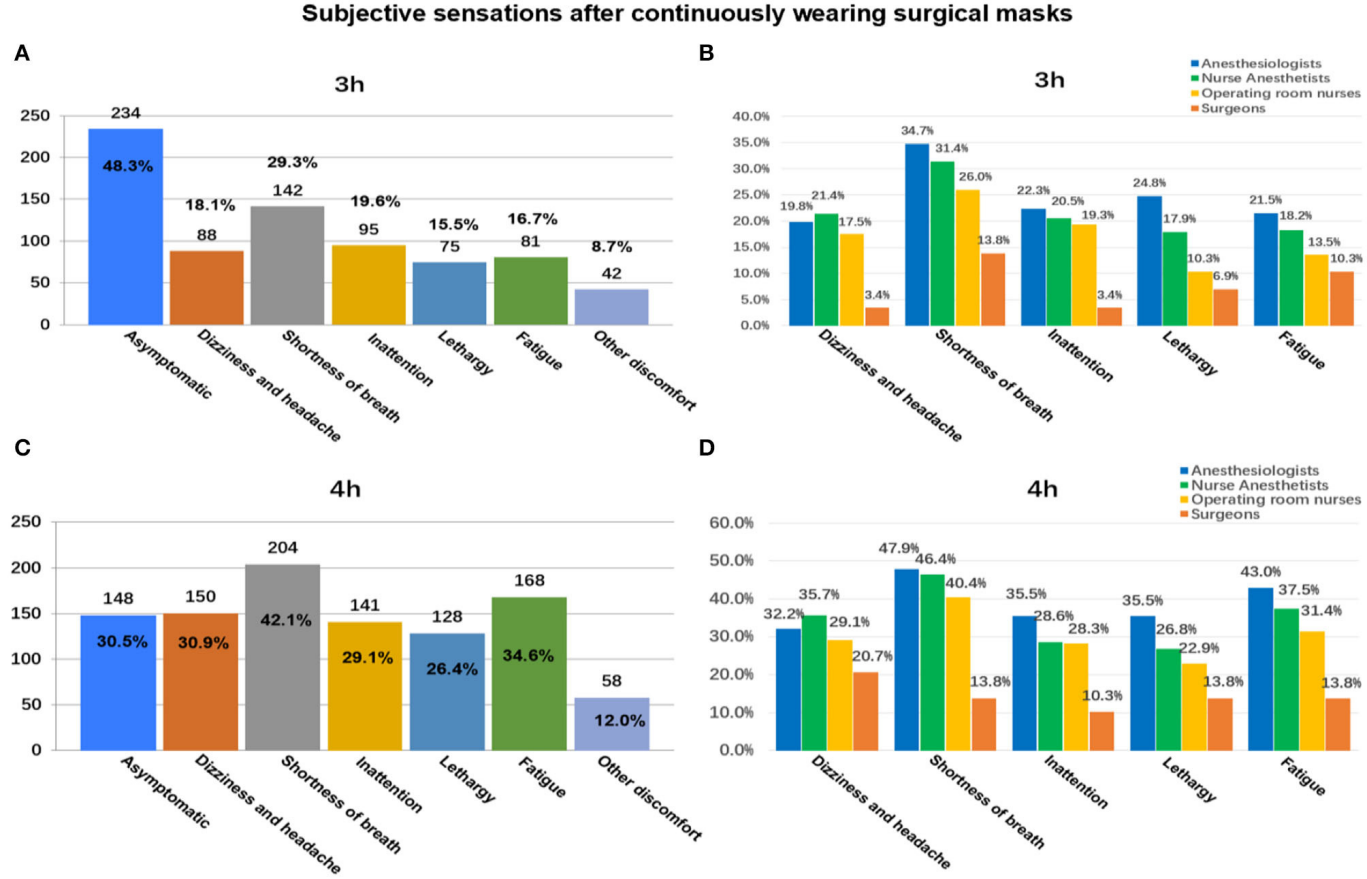
Subjective discomfort associated with continuously wearing surgical masks for 3 and ≥4 h: among all participants **(A,B)** and between different occupations **(C,D)**.

As shown in Figure 3A, 77.73% (*n* = 377/485) wore SMs continuously for 1 h without any subjective discomfort. As the SM use time increased, this proportion decreased significantly (Figures 3C, 4A,C). While wearing an SM for 4 h, only 30.52% (*n* = 148/485) had no discomfort (Figure 4C). With prolonged SM use, discomfort, such as shortness of breath, dizziness/headache, fatigue, and inattention, increased significantly. When wearing an SM continuously for 4 h, the proportions of shortness of breath, fatigue, and dizziness/headache reached 42.1% (*n* = 204/485), 34.6% (*n* = 168/485), and 30.9% (*n* = 150/485), respectively (Figure 4C). Compared with operating room nurses, nurse anesthesiologists, and surgeons, the proportions of subjective discomfort of anesthesiologists were almost all the highest in 1–4 h (Figures 3B,D, 4B,D).

### Basic Characteristics of Anesthesiologists and the Changes in Oxygen Content and Heart Rate After Different Durations of Mask Use

**Figure 5:**
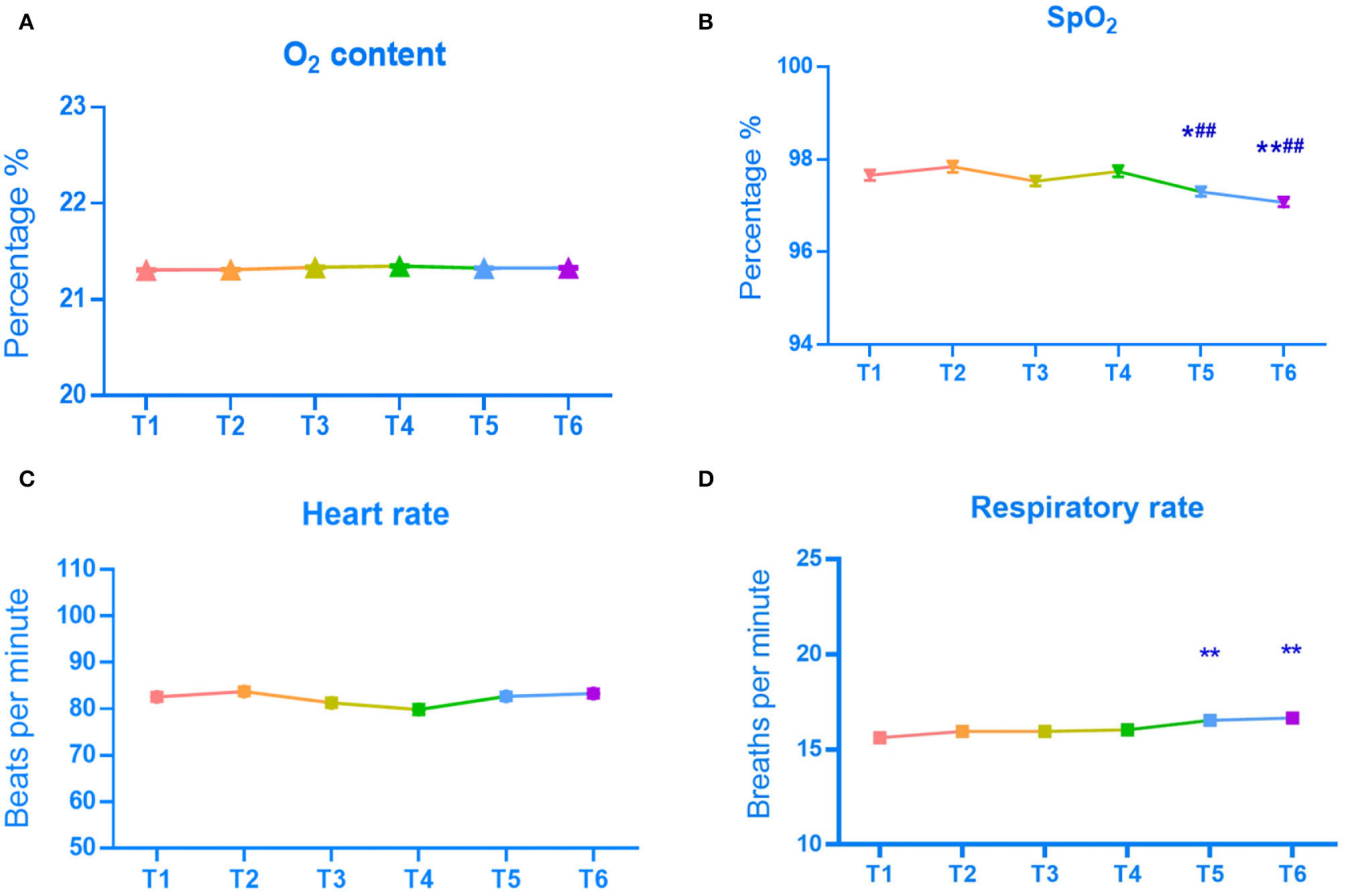
Mean O_2_ content **(A)**, SpO_2_ **(B)**, heart rate **(C)**, and respiratory rate **(D)** by time point. *P < 0.01, **P < 0.01, vs. the value of before SM use (T1). ##*P* < 0.01, vs. the value of immediately after SM use (T2).

In further research, 35 anesthesiologists (mean age, 34.5 years [SD, 7.9 years]; 16 men [46.0%]) were enrolled (Table 1). As shown in Figure 5A, there was no significant difference in the average oxygen content of the environment among time points. There was no significant difference in heart rate change at 5 time points after wearing a mask compared with that before use (Figure 5C).

**Table 1:** Baseline characteristics of participants.

**Characteristics**	**No. (%) of participants (***n*** = 35)**
Age, mean (SD), y	34.5 (7.9)
Gender
Men	16 (46)
Women	19 (54)
BMI, mean (SD), (kg m^−2^)	22.9 (3.3)

### Mean SpO_2_ and Respiratory Rate of Anesthesiologists by Time Point

The mean SpO_2_ from T1 to T6 was 98.0, 98.0, 98.0, 98.0, 97.0, and 97.0%, respectively (Table 2). As shown in Figures 5B,D, SpO_2_ gradually decreased, and the respiratory rate gradually increased as the duration of SM use increased. Compared with before and immediately after wearing an SM, after wearing an SM for 2 h, there was a statistically significant decrease in SpO_2_ (*p* < 0.05) and an increase in respiratory rate (*p* < 0.01).

**Table 2:** Results.

**Time points**	**O_2_ content (%)**	**SpO_2_ (%)**	**Heart rate**	**Respiratory rate**	**VAS score for shortness of breath**	**VAS score for dizziness and headache**
**T1**	21.3 (0.2)	98.0 (1.0)	85.0 (11.0)	16.0 (3.0)	0.0 (0.0)	0.0 (0.0)
**T2**	21.3 (0.2)	98.0 (1.0)	86.0 (12.0)	16.0 (2.0)	0.0 (0.0)	0.0 (0.0)
**T3**	21.4 (0.2)	98.0 (1.0)	82.0 (12.0)	16.0 (2.0)	0.0 (0.0)	0.0 (0.0)
**T4**	21.4 (0.1)	98.0 (1.0)	80.5 (13.0)	16.0 (2.0)	1.0**## (1.0)	1.0 **## (1.0)
**T5**	21.3 (0.2)	97.0*## (1.0)	84.0 (13.0)	17.0** (2.0)	1.0**## (1.0)	2.0**## (1.0)
**T6**	21.3 (0.2)	97.0 **## (1.0)	83.5 (13.0)	17.0 (2.0)**	2.0**## (2.0)	2.0**## (2.0)

*The data are presented as median (IQR) and Oxygen content, heart rate, SpO_2_, respiratory rate, and VAS scores by time point*.

* *p < 0.05*,

** *p < 0.01, vs. the value before SM use (T1)*.

## *p < 0.01, vs. the value immediately after SM use (T2)*.

### VAS Scores for Shortness of Breath, Dizziness, and Headache of Anesthesiologists by Time Point

**Figure 6:**
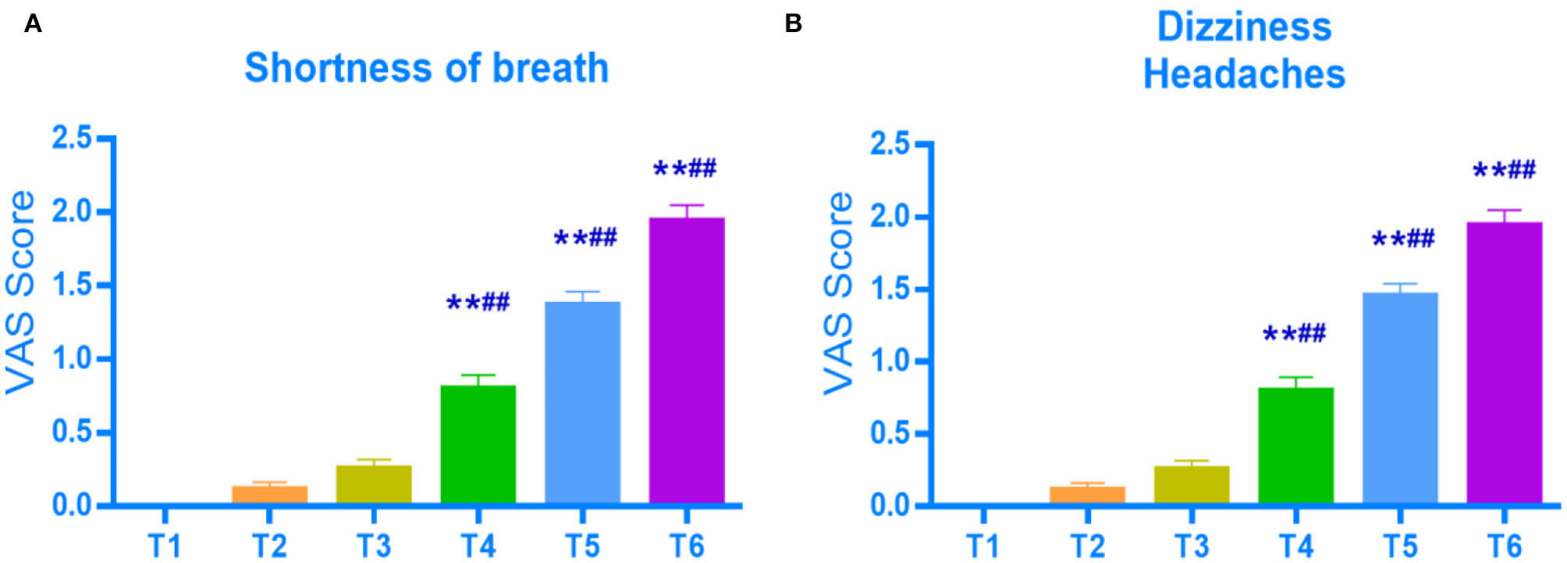
VAS scores for shortness of breath **(A)**, dizziness and headaches **(B)** by time point. **P < 0.01, vs. the value of before SM use (T1). ##*P* < 0.01, vs. the value of immediately after SM use (T2).

The VAS scores of 35 participants at six time points were compared. The results showed that the VAS scores for shortness of breath, dizziness, and headache increased significantly as the duration of continuous SM use increased (Table 2). Compared with before and immediately after wearing an SM, there was a significant difference in the VAS scores after wearing an SM ≥ 1 h (*p* < 0.01) (Figures 6A,B).

## Discussion

The results of the electronic questionnaire showed 70.5% of the respondents wore SMs from the beginning of a day's work to the end. Most staff (63.9%) continued to wear masks (without removing the mask or exposing their mouth or nose) for more than 4 h, with the highest proportion of anesthesiologists. Subjective discomfort, such as shortness of breath, dizziness and headache, inattention, lethargy, and fatigue, worsened with the prolonged use of SMs. Compared with operating room nurses, nurse anesthetists, and surgeons, with prolonged SM use, the proportion of subjective discomfort of anesthesiologists increased significantly, and was almost the highest in 1–4 h. Further research results showed that anesthesiologists wearing SMs for more than 2 h exhibited significantly reduced SpO_2_ levels and significantly increased respiratory rates, but no change was observed in heart rates. Although the data showed statistically significant differences, these changes have clinical significance needs to be further studied. In addition, wearing SMs for more than 2 h caused mild shortness of breath, dizziness, and headaches.

The results of this study are different from those of other studies. Many studies have shown that SMs can reduce oxygen concentrations and increase heart rate. Beder et al. repeatedly measured the SpO_2_ of 53 surgeons before and after surgery. The results showed that the surgeon's pulse rate increased, and their blood oxygen saturation decreased by more than 1% after 1 h, which was related to SM or operational stress (18). The SpO_2_ and heart rate of 20 oral surgeons wearing SMs during surgery were measured. It was found that as heart rate increased, SpO_2_ decreased from 97.5% before the operation to 94.0% after the operation, accompanied by shortness of breath and lightheadedness/headache (20). In a study of college students, it was found that the use of SMs in 150-min college courses could lead to an increase in heart rate and a decrease in blood oxygen saturation but it had no significant effect on students' perception of psychological fatigue or reaction time (21).

In this study, it was observed that the decrease in SpO_2_ and the increase in the respiratory rate occurred only after anesthesiologists wore SMs for more than 2 h, and it was observed that the degree of shortness of breath, dizziness, and headache increased as the duration of continuous mask use increased. However, no significant change in heart rate was observed, which may be because anesthesiologists who work in operating rooms and wear masks for long periods of time adapt to the psychological and physical effects caused by mask use and are familiar with the operating room environment. In addition, the long-term stress of anesthesiologists can aggravate occupational fatigue and lead to decreased reactivity and attention (22, 23), reducing the stress response of the cardiovascular system.

Wearing SMs in the operating room is not only a traditional practice (7, 24) but also a requirement for the prevention of surgical site infections (SSIs) and the protection of staff, according to the relevant guidelines of the Centers for Disease Control and Prevention (CDC) and the Healthcare Infection Control Practices Advisory Committee (HICPAC) (25). SMs are disposable and provide protection for at least 4 h (9), but this protective effect decreases over time. The general recommended time to wear an SM is 4 h (26). Studies have shown that SMs are a source of bacterial contamination during surgery. After 2 h of continuous SM use, the bacterial count in SMs increased significantly. Therefore, it is recommended that surgeons replace their masks after each operation, especially for operations lasting longer than 2 h (27). However, most operating room staff do not use masks correctly, as required, and do not understand the current guidelines for mask use, which may increase the SSI rate (28).

One study that used thermal infrared imaging was used to evaluate changes in facial skin temperature while wearing masks, and perceptual scores related to humidity, heat, dyspnea, and overall discomfort were recorded (16). It was found that wearing an SM or respirator continuously for 1 h leads to an increase in facial skin temperature under the mask and subjective discomfort, which decreases rapidly after removing the mask for 1 min and returns to the baseline level after 5 min (16). This study showed that heat stimulation on the surface around the mouth, nose, and cheeks plays an important role in regulating heat exchange in the respiratory tract. An earlier study showed that SMs may increase airway resistance and significantly reduce the surgeon's blood oxygen saturation level (29). A recent study also confirmed that airway resistance with SMs is twice as high as that without a mask, which causes an increase in heart rate (30).

The decrease in SpO_2_ may be due to the increased CO_2_ content in the inhaled air resulting from exhaled CO_2_ becoming trapped under the SM. While wearing an SM, repeated inhalation and exhalation of a small amount of CO_2_ may increase dyspnea (31). Mild CO_2_ retention and hypoxemia may also lead to cognitive effects and reduce responsiveness (32). A small decrease in oxygen stimulates the sympathetic nervous system, resulting in a faster heart rate (33). The increase in respiratory resistance caused by masks leads to an increase in respiratory muscle work and intrathoracic negative pressure, increases cardiopulmonary oxygen consumption, significantly increases heart rate, and further leads to physical discomfort and increased pressure (34). During the COVID-19 outbreak, medical staff had headaches due to the use of masks. In addition, symptoms, such as shortness of breath, sleep disorders, and fatigue, increased significantly (35, 36).

This study has some limitations: (1) The questionnaire was released in advance, and responses were required to be submitted 1 week after becoming familiar with the questions, which may introduce recall bias, and the subjects may have provided “ideal” answers. (2) The anesthesiologists involved in the study were younger and more tolerant of hypoxia caused by wearing SMs for a long time, so the time of oxygen saturation reduction and respiratory rate increase was longer. (3) There was no control group because the study was conducted in the emergency situation of the COVID-19 pandemic. According to the requirements of epidemic prevention and control and operating rooms, masks must be worn. (4) The sample size was too small to carry out age- and gender-controlled research.

In conclusion, our study demonstrates that in healthy anesthesiologists, wearing SMs for more than 2 h can lead to statistically significant decreases in SpO_2_ and increases in respiratory rates, without affecting heart rates, but may not be clinically significant. Shortness of breath, dizziness, and headache gradually worsen as the duration of SM use increases. Therefore, we recommend that anesthesiologists take off their masks and rest for a few minutes every 2 h or after each operation and replace the SM every 4 h according to the guidelines.

## Data Availability Statement

The original contributions presented in the study are included in the article/supplementary material, further inquiries can be directed to the corresponding author.

## Ethics Statement

The studies involving human participants were reviewed and approved by Qilu Hospital of Shandong University Medical Ethics Committee. Written informed consent for participation was not required for this study in accordance with the national legislation and the institutional requirements.

## Author Contributions

XL, CF, SY, and RW contributed to data collection. CF, YL, SY, and SH contributed to statistical analysis. SY, FQ, and YL wrote the manuscript. SY, XL, FQ, and CF contributed to the inception of the study idea. SY, XL, FQ, and RW contributed to study design. SY, FQ, YL, and SH revised the manuscript. All authors approved the final manuscript.

## Conflict of Interest

The authors declare that the research was conducted in the absence of any commercial or financial relationships that could be construed as a potential conflict of interest.

## Publisher's Note

All claims expressed in this article are solely those of the authors and do not necessarily represent those of their affiliated organizations, or those of the publisher, the editors and the reviewers. Any product that may be evaluated in this article, or claim that may be made by its manufacturer, is not guaranteed or endorsed by the publisher.
